# Subcellular Localization of MxB Determines Its Antiviral Potential against Influenza A Virus

**DOI:** 10.1128/JVI.00125-20

**Published:** 2020-10-27

**Authors:** Fiona Steiner, Jovan Pavlovic

**Affiliations:** a Institute of Medical Virology, University of Zurich, Zürich, Switzerland; Icahn School of Medicine at Mount Sinai

**Keywords:** Mx proteins, MxB, influenza A viruses, interferon system, subcellular localization, viral specificity

## Abstract

Mx proteins are interferon (IFN) type I (α/β)- and type III (λ)-induced effector proteins with intrinsic antiviral activity. Mammalian Mx proteins show different subcellular localizations and distinct yet partially overlapping viral specificities. However, the precise mechanism(s) of antiviral action are still unresolved. Human MxA accumulates in the cytoplasm and inhibits a wide variety of RNA and DNA viruses, among them influenza A virus (IAV). In contrast, MxB, the second human Mx protein, localizes via its amino (N) terminus to the outer nuclear membrane at or near nuclear pores and inhibits the nuclear import of incoming human immunodeficiency viruses (HIV) and herpesviruses, but not that of IAV. Here, we evaluated whether the antiviral specificity of MxB is determined by its subcellular localization. For this purpose, we redirected MxB to the nucleus or cytoplasm by either attaching a nuclear localization signal to its N terminus or by exchanging the N terminus of MxB with that of MxA. Interestingly, ectopic expression of these MxB variants in the nucleus or in the cytoplasm rendered the host cells resistant to IAV, revealing that the capacity of MxB to block IAV replication critically depends on the site where the protein accumulates in the infected cell. Furthermore, coimmunoprecipitation (co-IP) assays demonstrated that MxB physically interacted with the nucleoprotein (NP) of IAV. Taken together, the data indicate that the subcellular localization of the MxB protein plays a pivotal role in determining its antiviral specificity.

**IMPORTANCE** The interferon system plays a pivotal role in the defense against viral infections. The dynamin-related Mx proteins form a small family of interferon-induced effector proteins with distinct antiviral specificities and subcellular localizations. So far, it is not clear whether the different virus specificities of Mx proteins are the result of distinct mechanisms of action or are due rather to their different subcellular localization. We show here that the human MxB protein, normally localized to the outer membrane of the cell nucleus, acquires antiviral activity against IAV when redirected to the nucleus or cytoplasm, subcellular sites where other members of the Mx protein family efficiently interfere with IAV replication. Our findings thus strongly suggest that Mx proteins act primarily through a common mechanism and that their viral specificity is at least in part determined by their individual subcellular localization.

## Abstract (precis)

The interferon system plays a pivotal role in the defense against viral infections. The dynamin-related Mx proteins form a small family of interferon-induced effector proteins with distinct antiviral specificities and subcellular localizations. So far, it is not clear whether the different virus specificities of Mx proteins are the result of distinct mechanisms of action or are due rather to their different subcellular localization. We show here that the human MxB protein, normally localized to the outer membrane of the cell nucleus, acquires antiviral activity against IAV when redirected to the nucleus or cytoplasm, subcellular sites where other members of the Mx protein family efficiently interfere with IAV replication. Our findings thus strongly suggest that Mx proteins act primarily through a common mechanism and that their viral specificity is at least in part determined by their individual subcellular localization.

## INTRODUCTION

Mx proteins are dynamin-related GTPases that play key roles in the interferon type I- and type III-mediated innate immune response against a variety of viruses (reviewed in references 1 and 2). They interfere with the replication of many RNA and DNA viruses by inhibiting early steps of their life cycle. The two MX genes (*MX1* and *MX2*) of the human genome encode the two proteins MxA and MxB (also termed Mx1 and Mx2). The MxA protein restricts a broad spectrum of viruses, primarily negative-stranded RNA viruses, but also some positive-stranded RNA viruses and DNA viruses (for a review, see reference 3). The subcellular localization determines in part the antiviral specificity of Mx proteins (4, 5). MxB has recently been shown to inhibit the replication of primate (nonhuman and human) lentiviruses, herpesviruses, and cyclophilin A-dependent flaviviruses (6–12). Mammalian Mx proteins accumulate in different subcellular compartments. Human MxA accumulates in the cytoplasm and has been reported to be partly associated with the plasma membrane, as well as with the smooth endoplasmic reticulum, but it can also be found in metastable membraneless cytoplasmic condensates and was shown to form inflammasomes in cells of the respiratory epithelium (13–16). MxB exists in at least two isoforms of 715 and 690 amino acids. The longer isoform represents the full-length form, which includes an N-terminal nuclear localization signal (NLS), while the shorter isoform is translated from a downstream translation start site and lacks the NLS (17, 18). The full-length MxB is localized primarily on the cytoplasmic face of the nuclear membranes, but when expressed ectopically it can also be found in the cytoplasm or in the nucleus in a granular pattern in the heterochromatin region beneath the nuclear envelope (6, 18, 19). The short isoform is expressed in the cytoplasm (20, 21) and does not exhibit any known antiviral activity (6, 11, 22).

The mouse genome carries two Mx genes encoding nuclear Mx1 and cytoplasmic Mx2 (23, 24). Mouse Mx1 protein contains an NLS in the carboxy (C)-terminal region and is thus exclusively expressed in the cell nucleus (24, 25). Mouse Mx1 inhibits members of the orthomyxovirus family, including influenza A virus (IAV), which replicates in the cell nucleus. In contrast, Mx2 restricts vesicular stomatitis virus (VSV) and hantavirus, both negative-stranded RNA viruses that replicate in the cytoplasm (4, 23, 26). Intriguingly, human MxA restricts not only various RNA viruses replicating in the cytoplasm such as VSV, La Crosse virus, or Semliki forest virus (SFV), but also several members of the orthomyxoviruses, including IAV (reviewed in references 1 and 3). Mx proteins require a functional G domain to inhibit Mx-sensitive viruses; exceptions are MxB when targeting lentiviruses (7) and MxA blocking hepatitis B virus (HBV) (27). Although the viral targets of Mx proteins are known for many Mx-sensitive viruses to be nucleoproteins, viral ribonucleoprotein (vRNP) complexes or nucleocapsids (reviewed in references 1 and 3), the molecular mechanism(s) of action of Mx proteins remain to be elucidated. In the case of IAV, the prevailing model predicts that Mx1 as well as MxA attach to the IAV vRNPs by forming oligomeric “ring-like” structures, thereby blocking the activity of the viral polymerase (28–30). However, this mode of action does not explain the pronounced inhibition of the IAV based minireplicon system, in which replication occurs from vRNPs formed in the nucleus (31). Hence, MxA must act at an additional step of the viral replication cycle. MxA is thought to exert a gatekeeper function in the cytoplasm, blocking or modifying NP of incoming vRNPs and/or newly synthesized NP in the cytoplasm of infected cells. Similarly, for MxB, evidence also points to a gatekeeper function by preventing the nuclear import of genomic DNA of human lentiviruses and herpesviruses into the nucleus (2, 6, 12). In addition, Lee and colleagues have recently shown that MxA also acts as an inflammasome sensor protein in human respiratory epithelial cells by recognizing IAV NP, thereby triggering inflammasome formation and interleukin 1β (IL-1β) secretion (15).

The fact that Mx proteins inhibit a wide variety of RNA and DNA viruses at different subcellular locations raises the question of whether (i) Mx proteins act via a largely common mechanism and the observed virus specificity is a consequence of distinct subcellular localization or (ii) Mx proteins act through different specific molecular mechanisms.

To address these questions, we made use of human MxB predominantly accumulating in the perinuclear region. This protein exerts antiviral activity against HIV and herpesviruses but not against IAV (32). We reasoned that if the hypothesis of a common antiviral mechanism between MxA and MxB was correct, a nuclear or cytoplasmic form of MxB should also restrict IAV replication. We show here that MxB retargeted to either the nucleus or the cytoplasm via a canonical NLS or the N-terminal end of MxA indeed blocked IAV replication. Intriguingly, MxB retargeted to the nucleus still efficiently blocked HIV-1 infection, suggesting that MxB is able to block HIV at different steps of its life cycle. These data underline the importance of the subcellular localization of Mx proteins and suggest that Mx proteins act through a common mechanism.

## RESULTS

**FIG 1:**
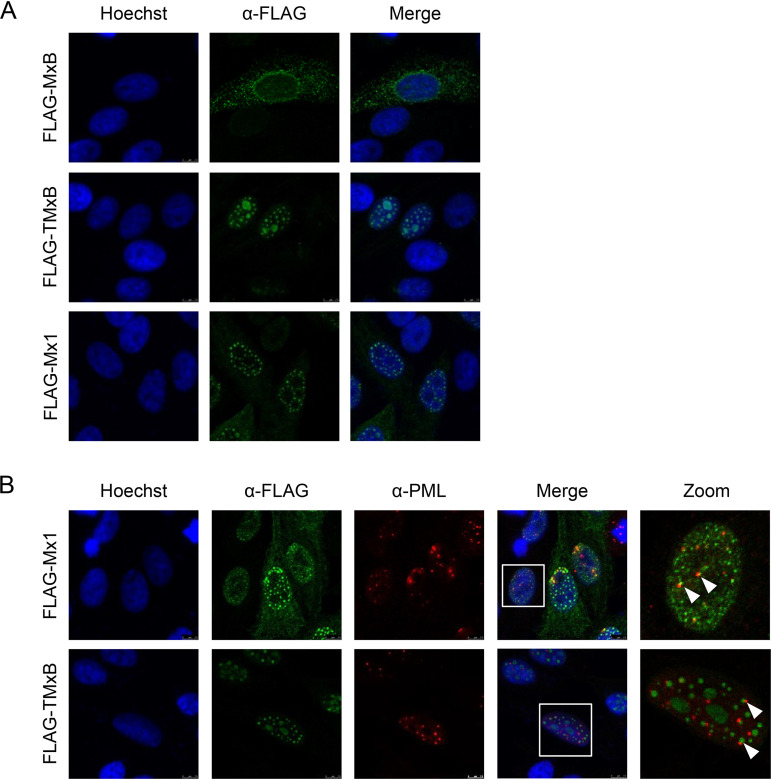
TMxB accumulates in the cell nucleus and is associated with promyelocytic leukemia (PML) bodies. (A) HEp-2 cells were transfected with plasmids coding for FLAG-tagged wild-type (wt) MxB (MxB wt), FLAG-tagged MxB containing an N-terminal nuclear localization signal (NLS), (TMxB), and FLAG-tagged wild-type mouse Mx1 (Mx1 wt). Cells were fixed and immune stained with a mouse monoclonal antibody specific for the FLAG tag. Images are representative for two independent experiments. (B) HEp-2 cells were transfected with plasmids coding for nuclear MxB (TMxB) and wild-type Mx1. Cells were fixed and immune stained with a mouse monoclonal antibody specific for the FLAG tag and with a rabbit polyclonal PML antibody. PML bodies, promyelocytic leukemia bodies in the nucleus. Arrows depict examples of TMxB-PML body in juxtaposition.

We first tested whether full-length MxB containing the NLS of the simian virus 40 (SV40) large T antigen at its N terminus (designated TMxB) would be expressed in the cell nucleus. We transiently transfected HEp-2 cells with plasmids coding for FLAG-tagged MxB, TMxB, and mouse Mx1 accumulating in the cell nucleus (24). The SV40-NLS was attached to the N terminus of MxB since Mx proteins with modifications at their C-terminal ends (deletions or tags) lack GTPase activity and hence antiviral activity against IAV (4, 5, 33). As expected, TMxB accumulated exclusively in the nucleus, showing different size speckles and a weak staining throughout the nucleus. Mx1 showed a similar punctuated pattern in the nucleus, omitting the nucleoli (Fig. 1A). Since Mx1 assemblies have often been observed to be distributed in juxtaposition to promyelocytic leukemia (PML) bodies in the nucleus (34, 35), we tested whether this is also the case for TMxB. For this purpose, we cotransfected HEp-2 cells with plasmids encoding FLAG-tagged TMxB, or mouse Mx1 and immunostained PML bodies and the FLAG peptide in parallel. Indeed, TMxB assemblies were often found next to PML bodies (Fig. 1B), suggesting that the TMxB speckles were not randomly distributed in the cell nucleus but were localized to distinct subnuclear structures similar to those of Mx1 (35).

**FIG 2:**
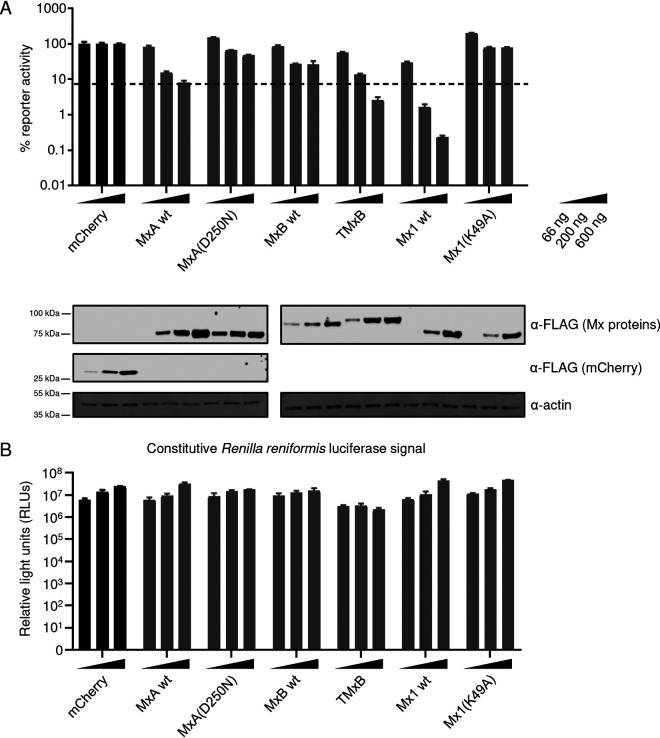
The nuclear variant of MxB (TMxB) exhibits restriction of IAV replication. (A) Minireplicon assay of 293T cells transfected with plasmids encoding the minireplicon constituents and increasing amounts of FLAG-mCherry or the indicated FLAG-Mx proteins. Cells were lysed and a dual-luciferase assay was performed. Values are presented in percent firefly luciferase activity normalized to *Renilla* luciferase relative to the mCherry control for each individual condition (66 ng, 200 ng, and 600 ng). Values of mCherry-expressing samples are set to 100%. Data are represented as mean ± standard deviation (SD) of triplicates. The experiment was performed in biological triplicates. Western blot analysis was carried out with pooled lysates for each condition, and immunostaining was performed with the indicated antibodies (bottom). (B) *Renilla* luciferase activity in the minireplicon assays shown in panel A. Data are represented as mean ± SD of triplicates.

Next, we tested whether TMxB, by virtue of its nuclear localization, inhibits IAV replication utilizing the IAV Kan1-based minireplicon system (36). For this purpose, HEK293T cells were cotransfected with increasing amounts of plasmids (66 ng, 200 ng, and 600 ng) encoding either mCherry (negative control), wild-type (wt) MxA, the inactive G-domain mutant MxA(D250N), wild-type MxB, TMxB, wild-type mouse Mx1, and the inactive G-domain mutant Mx1(K49A) together with plasmids encoding PB1, PB2, PA, and NP of the A/Thailand/1(KAN-1)/2004 (H5N1) strain, the firefly luciferase reporter, and *Renilla* luciferase under a constitutive promoter. The G-domain mutants of MxA and Mx1, previously shown to be inactive, were included as negative controls (37, 38). Intriguingly, TMxB restricted IAV replication at least as strongly as MxA, whereas MxB showed only marginal activity at high concentrations of the transgene (Fig. 2A). As expected, Mx1 showed a strong inhibition of IAV, whereas the G-domain mutants MxA(D250N) and Mx1(K49A) exhibited no inhibitory activity. The activity of *Renilla reniformis* luciferase expressed under the control of an constitutive promoter varied little and was not influenced by the Mx variant expressed, demonstrating that none of the Mx variants exerted an unspecific inhibitory effect (Fig. 2B) Hence, this experiment clearly demonstrates that MxB has the capacity to restrict the replication of IAV when translocated to the nucleus.

**FIG 3:**
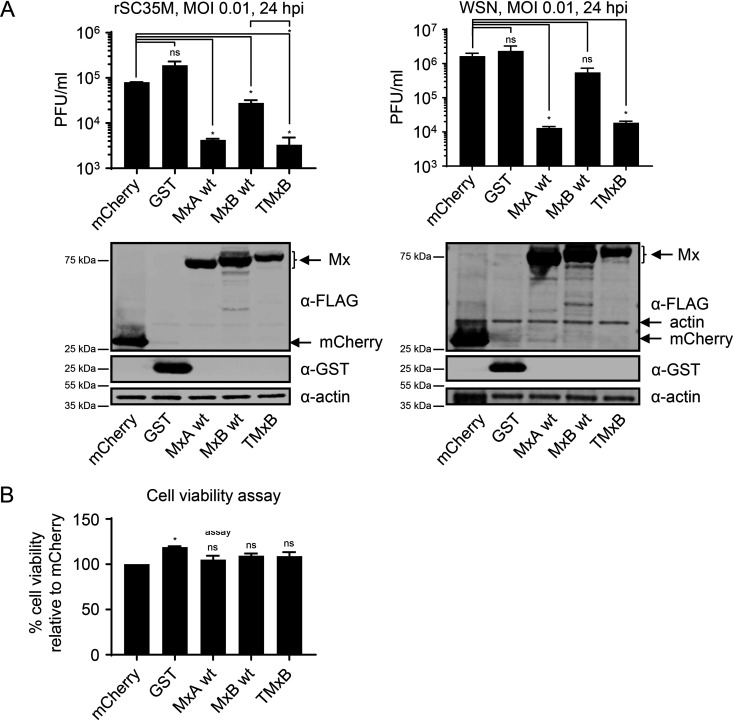
TMxB blocks IAV infection. (A) Plaque assay titration of supernatants harvested from 293T cells transiently transfected with plasmids coding for the indicated control protein or FLAG-Mx proteins and infected with a multiplicity of infection (MOI) of 0.01 for 24 h. Supernatants were titrated on MDCK cells. Data are represented as mean ± standard error of the mean (SEM) of duplicates. Bottom panels show expression control. Samples were harvested at the same time as supernatants were collected for the plaque assay. Western blot analysis was performed using the indicated antibodies (bottom panel). Data are representative for two independent experiments. (B) CellTiter-Glo cell viability assay. 293T cells were transfected as in panel A and cells were lysed at the same time as supernatant was collected for the plaque assay from an identical plate. Luminescence was measured, and absolute values were plotted. Data are represented as mean ± SEM of duplicates. *P* < 0.05; Student’s *t* test.

In order to corroborate the antiviral potential of TMxB against IAV, we tested whether TMxB would also exert anti-influenza activity in the context of infection. We transfected HEK293T cells with plasmids encoding wild-type MxB, TMxB, and MxA or control plasmids coding for mCherry or glutathione *S*-transferase (GST), and infected the cells at 24 h posttransfection with 0.01 MOI of the IAV strains A/Seal/Massachusetts/1/1980 (H7N7, designated rSC35M) or A/WSN/1933(H1N1). At 24 h postinfection, the culture supernatants were harvested and subjected to plaque assay analysis (Fig. 3). These data clearly indicate that TMxB expression restricted IAV to a similar extent as MxA, resulting in a 12-fold reduction of rSC35M titers and a 90-fold reduction of WSN titers. Cell viability assays performed in parallel revealed that the observed reduction in titers was not due to cytotoxic effects of TMxB expression (Fig. 3B). Furthermore, expression levels of the three Mx transgenes were comparable (Fig. 3A, lower panels).

**FIG 4:**
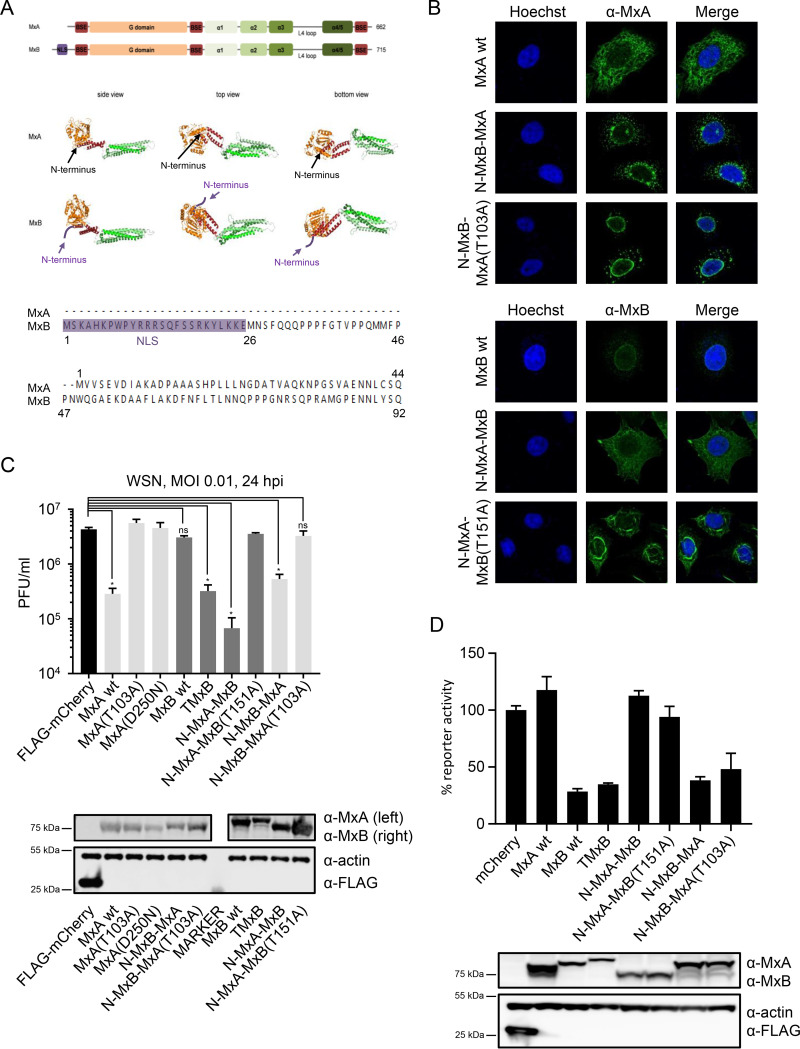
MxB with the N terminus of MxA relocalizes to the cytoplasm and acquires anti-IAV activity. (A) Top: graphical representation of MxA and MxB. Middle: crystal structure of MxA (28-amino-acid deletion in loop L4; PDB identifier [ID] 3SZR) and MxB (83-amino-acid deletion at the N terminus; PDB ID 4WHJ). Bottom: alignment of the amino acid sequence of the N termini of MxA and MxB. (B) Subcellular localization of MxA-MxB chimera. HEp-2 cells were transfected with plasmids expressing the indicated MxA or MxB variants or chimera. Fixed cells were immunostained with a monoclonal mouse anti-MxA or anti-MxB antibody. Shown are representative images of 2 independent experiments. (C) IAV replication capacity in the presence of MxA, MxB, TMxB, and the indicated MxA-MxB chimera. 293T cells were transiently transfected with plasmids coding for the indicated proteins for 24 h and were subsequently infected with WSN at an MOI of 0.01 for 24 h. Protein expression was controlled by immunoblot analysis. Supernatant was transferred to MDCK cells and viral titers were determined 24 h postinfection. Data are representative for three independent experiments. Data are represented as mean ± SD of triplicates. *P* < 0.0001; Student’s *t* test. (D) HIV-1 reporter virus replication capacity in the presence of MxA, MxB, TMxB, and the indicated MxA-MxB chimera. HeLa cells were transiently transfected with expression plasmids coding for the indicated proteins. At 24 h posttransfection, cells were infected with VSV-G-pseudotyped HIV-1 NL-Luc reporter virus. Luciferase assay was performed at 48 h postinfection, and reporter activity is shown relative to mCherry. Data are represented as mean ± SD of duplicates. Data are representative for three independent experiments.

Thus, we could show that relocalization of MxB to the nucleus is essential for IAV restriction. In the next step, we wanted to test whether relocalization of MxB to the cytoplasm would also lead to a gain of antiviral activity against IAV. The signal element of MxB targeting it to the outer surface of the nuclear envelope is located in the unstructured region at the very N terminus of the protein (22), just preceding the bundle signaling element (BSE) and GTPase domain (Fig. 4A). The N-terminal end of MxB is not included in the schematic drawing, since it was deleted for crystallization due to its unstructured nature (17). This is also the region where major sequence differences between MxA and MxB are observed (39). For the long isoform of MxB (perinuclear localization), this N-terminal region comprises 91 amino acids, whereas in MxA only 43 amino acids are present (Fig. 4A). In order to alter the subcellular localization and thus possibly the antiviral specificity, we generated two types of MxA-MxB chimeras with interchanged N termini, namely, MxB(1-91)-MxA(44-662) (designated N-MxB-MxA) and MxA(1-43)-MxB(92-715) (designated N-MxA-MxB). Localization was monitored by immunofluorescence analyses in transfected cells (Fig. 4B). Proteins harboring the MxB N terminus localized to the nuclear envelope and were additionally seen in distinct punctate pattern in the cytoplasm as previously observed (22). Remarkably, the N-MxB-MxA chimera showed only a loose association with the nuclear envelope and localized predominantly in a punctate pattern in the perinuclear region, while its GTPase-deficient counterpart N-MxB-MxA(T103A) was strongly associated with the nuclear envelope (Fig. 4B). On the other hand, ectopic expression of N-MxA-MxB exhibited the typical cytoplasmic distribution of MxA, whereas the GTPase-deficient variant N-MxA-MxB(T151A) showed primarily a strong rod-like perinuclear staining. Intriguingly, destruction of the GTPase activity appears to influence the subcellular localization of the chimeric MxA-MxB proteins.

To assess the antiviral potential of these chimeras, we again performed viral plaque assays as described above. In addition to the anti-IAV activity of TMxB (13-fold inhibition), chimeric N-MxA-MxB also showed potent IAV restriction (42-fold inhibition) (Fig. 4C). N-MxB-MxA exhibited a less pronounced but still significant activity (8-fold reduction), again suggesting that the subcellular localization does play a crucial role. The viral restriction was completely abrogated when the GTPase activity of these chimeras was destroyed by introducing mutations into their G-domain (T103A for MxA and T151A for MxB) (Fig. 4C). These data indicate that MxB exerts its anti-IAV activity by a similar molecular mechanism to that of MxA, whose anti-IAV activity is strictly dependent on its GTPase activity (37).

Goujon and colleagues have previously shown that the N-MxB-MxA chimera accumulating in the perinuclear region efficiently restricted HIV infection, whereas the cytoplasmic N-MxA-MxB variant showed no anti-HIV activity (22), These findings demonstrated that there is a clear correlation between subcellular localization of MxB and its anti-HIV activity (22). Therefore, we expected that TMxB residing exclusively in the cell nucleus would lose anti-HIV activity. To test this hypothesis, we transfected HeLa cells with plasmids coding for MxA, MxB, TMxB, N-MxA-MxB, N-MxB-MxA, and the GTPase-deficient variants N-MxA-MxB(T151A) or N-MxB-MxA(T103A) and subsequently transduced them with a VSV-G-pseudotyped HIV-green fluorescent protein (GFP) reporter virus. Surprisingly, TMxB exhibited a strong anti-HIV activity similar to that of MxB, N-MxB-MxA, and its GTP-deficient variant (Fig. 4D). As expected, the mCherry control, MxA, and the N-MxA-MxB variants did not restrict HIV (Fig. 4D). Hence, the data corroborate the suggestion that MxB has the capacity to restrict HIV at multiple steps (40, 41).

**FIG 5:**
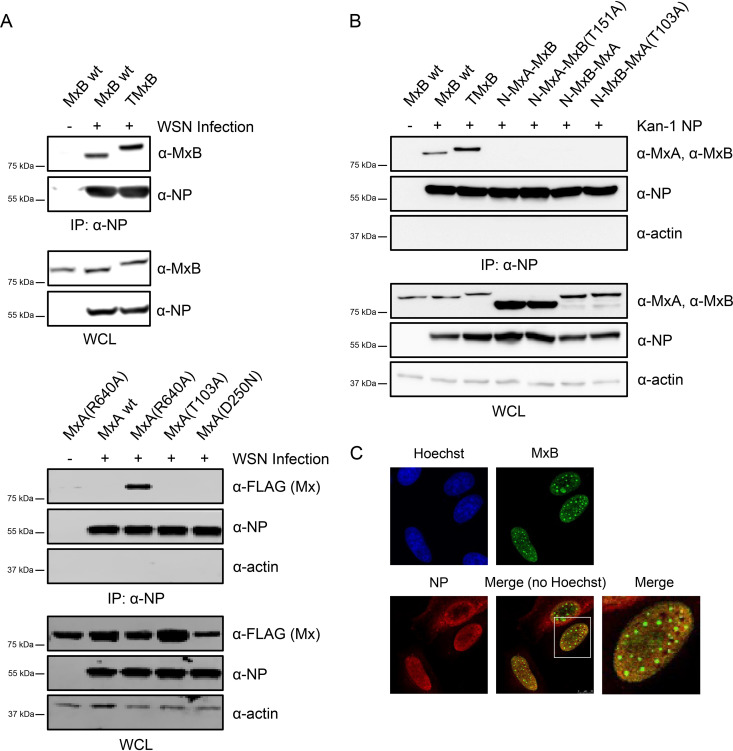
MxB forms a stable complex with NP. (A) HeLa cells were transfected with plasmids encoding the indicated Mx proteins. At 24 h posttransfection, cells were infected with WSN at an MOI of 5 for 6 h. Cells were lysed, and viral NP was immunoprecipitated (IP) from lysates using a mouse monoclonal anti-NP antibody. The resulting complexes were subjected to Western blot analysis using the indicated antibodies. In addition, whole-cell lysates (WCL) were also monitored for expression of Mx, NP, and actin by Western blot analysis. (B) HEp-2 cells were transfected with plasmids coding for the indicated Mx proteins and either a Kan1 NP-expressing plasmid or an empty vector. At 24 h posttransfection, cells were lysed and the IP was performed as in panel A. (C) HEp-2 cells were transfected with plasmids coding for FLAG-TMxB and WSN NP. Cells were fixed and stained with a rabbit antibody specific for the FLAG tag and a mouse monoclonal antibody specific for IAV NP.

Although many studies have demonstrated that MxA targets NP, a direct interaction of MxA with NP has not been observed so far (42–44). The general assumption is that oligomeric MxA only transiently interacts with NP to exert its anti-IAV function. However, the dimeric mutants of MxA [like MxA(R640)] are able to form stable complexes with NP (44). Since MxB appears to exist predominantly as a dimer (17), we next tested whether MxB has also the capacity to form stable complexes with NP. For this purpose, we transiently transfected HEp-2 cells with plasmids encoding MxB wt and TMxB. As controls, we included plasmids coding for MxA wt, MxA(R640A), and the two GTPase-deficient MxA mutants MxA(T103A) and MxA(D250N). At 24 h posttransfection, the cells were infected with WSN at an MOI of 5 for 6 h, and the lysates were subjected to coimmunoprecipitation (co-IP) experiments using a monoclonal mouse anti-NP antibody (Fig. 5A). Surprisingly, wild-type MxB, as well as TMxB, efficiently coprecipitated with viral NP, indicating that MxB has indeed the capacity to form stable complexes with NP, although independently of its subcellular localization (Fig. 5A). Hence, the capacity of MxB to form a complex with NP is not sufficient for its anti-IAV activity, and other parameters like stoichiometry and additional interactors appear to play a crucial role. As expected, MxA(R640A) formed stable complexes with viral NP, while MxA wt and the antivirally inactive GTPase-deficient mutants MxA(T103A) and MxA(D250N) did not coprecipitate with NP.

Next, we tested whether the MxA-MxB chimera would also form stable complexes with NP. To this end, we cotransfected plasmids encoding MxB wt, TMxB, N-MxA-MxB, N-MxB-MxA, and their GTPase deficient variants NMxA-MxB(T151A) and N-MxB-MxA(T103A) with an expression plasmid coding for the NP of the avian Kan-1 strain (Fig. 5B). The data show that MxB and TMxB were efficiently coprecipitated with NP, indicating that MxB has the capacity to form stable complexes with ectopically expressed NP in the absence of other viral proteins. In contrast, irrespective of their anti-IAV activity, none of the MxA-MxB chimeras coprecipitated with NP, again emphasizing the notion that for Mx proteins the formation of stable complexes with NP is not a prerequisite for anti-IAV function.

Next, we tested whether the stable complexes between TMxB and NP could also be observed microscopically. For this purpose, we cotransfected HEp-2 cells with expression plasmids coding for FLAG-TMxB and NP (WSN). The cells were fixed at 24 h posttransfection and subjected to immunostaining with antibodies specific for the FLAG tag and for NP. The data show colocalization (yellow staining) of TMxB and NP throughout the nucleus, with the exception of the TMxB assemblies. This finding suggests that colocalization and thus potential interaction of TMxB and NP occurs predominantly at regions were TMxB is evenly distributed and not in the form of large aggregates (Fig. 5C).

**FIG 6:**
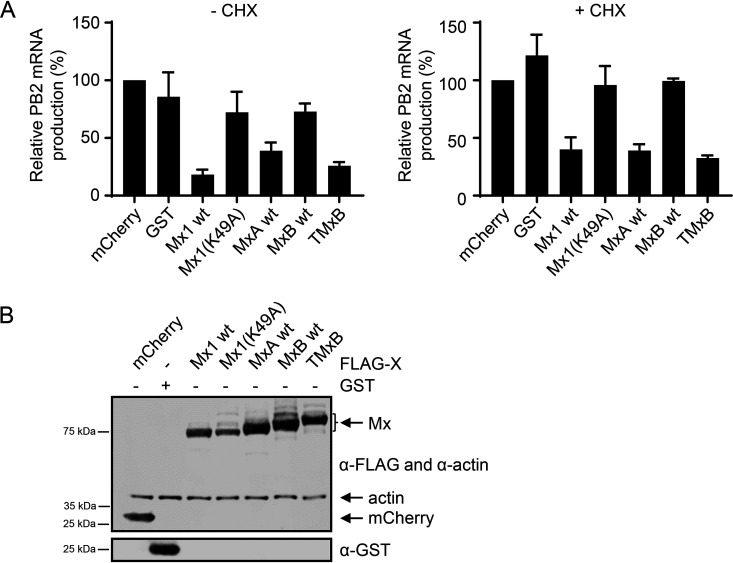
Primary transcription of IAV is inhibited by TMxB. (A) 293T cells were transfected with plasmids coding for the indicated control or FLAG-Mx proteins and infected with rSC35M(H7N7) at an MOI of 5 for 6 h. RNA was isolated and reverse transcribed into cDNA using random primers. RT-qPCR analysis was performed, and PB2 mRNA normalized to GAPDH mRNA is shown. Cells were pretreated with 100 μg/ml cycloheximide (CHX) or with the same volume of solvent (DMSO) for 1 h prior to infection. Where indicated, infection was carried out in the presence of CHX. Values are relative to the mCherry control, which was set to 100%. Data are represented as mean ± SEM of triplicates. (B) Western blot analysis of 293T cells transfected and lysed at the same time as those in panel A. Immunostaining was performed with the indicated antibodies.

Since Mx proteins such as Mx1 and TMxA, which localize to the nucleus, inhibit IAV replication at or before the primary transcription of viral mRNAs (4), we tested whether this also applies for TMxB. We transiently transfected HEK293T cells with plasmids encoding mCherry, GST, Mx1, the G-domain mutant Mx1(K49A), MxA, MxB, or TMxB and infected the cells with an MOI of 5 of the avian strain rSC35M for 6 h in the presence or absence of cycloheximide (CHX). In the presence of cycloheximide IAV, vRNPs are still able to enter the nucleus and to produce viral transcripts by the vRNP-associated viral polymerase. However, later steps are blocked by the translational inhibition exerted by cycloheximide (45). Overall mRNA levels were thus reduced in CHX-treated versus dimethyl sulfoxide (DMSO)-treated samples since the viral proteins PB1, PB1, PA, and NP, which are crucial for viral genome replication, are not translated. As expected, viral mRNA levels were 35- to 100-fold lower in CHX-treated samples compared to those in DMSO-treated controls (data not shown). Synthesis of viral mRNA was determined by reverse transcription-quantitative PCR (RT-qPCR) with primers specific for PB2. Indeed, TMxB inhibited viral RNA synthesis at a step prior to or during primary transcription to a very similar extent as that of Mx1 (Fig. 6). As expected, Mx1(K49A), which lacked a functional G domain, failed to inhibit viral mRNA synthesis in the presence or absence of cycloheximide (Fig. 6).

## DISCUSSION

The fact that Mx proteins, specifically MxA, are able to exhibit a very broad antiviral specificity, inhibiting many RNA as well as DNA viruses at different steps of their replication cycle, suggests that Mx proteins are multifunctional proteins that exert different strategies to block viral replication. For instance, MxA blocks the replication of viruses as diverse as VSV and HBV at different steps of their replication cycle (reviewed in references 1 and 3). Moreover, Mx proteins can be found in different subcellular compartments. Human MxA resides in the cytoplasm, where it is partially associated with internal membranes, particularly with the smooth endoplasmic reticulum, whereas the human MxB accumulates primarily in the perinuclear region associated with the outer membrane of the nucleus but can also be found in the cytoplasm (6, 13, 14, 18).

In this study, we addressed the question whether the apparent diversity of antiviral activities of Mx proteins is a consequence of distinct subcellular localizations rather than of a multitude of molecular mechanisms. To test this hypothesis, we employed the human MxB protein with well-defined antiviral activities against HIV, HSV, and cyclophilin A-dependent flaviviruses (7–9, 11, 26, 27) but not against IAV (2, 6, 11). We reasoned that redirecting MxB into the nucleus by attaching a classical NLS (termed TMxB) should enable it to block the replication of IAV, which occurs in the nucleus. The fact that we often found TMxB assemblies in juxtaposition with PML bodies, a feature previously noted for Mx1, strongly suggests that TMxB accumulates in distinct nuclear domains (Fig. 1B) (34, 35). Indeed, ectopic expression of TMxB but not of MxB efficiently blocked the expression of a luciferase reporter gene driven by the minireplicon system based on the avian IAV strain Kan1, indicating that MxB has the capacity to interfere with IAV replication when located in the nuclear compartment (Fig. 2). Moreover, TMxB also strongly restricted infection by IAV strains of avian (rSC35M), as well as that by a human-origin strain (WSN) (Fig. 3).

If the subcellular localization of the MxB protein indeed represented a major determinant of its antiviral specificity, MxB should also exert anti-IAV activity in the cytoplasm. Ectopic expression of a chimeric MxA(1-43)-MxB(92-715) protein containing the N-terminal 43 amino acids of MxA in place of the N-terminal 91 amino acids of MxB (Fig. 4A) revealed that this is indeed the case. This MxA/MxB chimera localized throughout the cytoplasm as wild-type MxA and very efficiently restricted WSN replication (Fig. 4B and C). The anti-IAV activity of this protein was strictly dependent on its GTPase activity, indicating that the observed restriction was indeed the result of the activity of MxB. Interestingly, the reverse chimera MxB(1-91)-MxA(44-662) containing the 91 N-terminal amino acids of MxB also exhibited a partial anti-IAV, activity as previously shown (22) (Fig. 4C). This restriction is also dependent on the GTPase activity of this protein (Fig. 4C). However, immunofluorescence imaging revealed that this chimera was less tightly associated with the nuclear envelope than was wild-type MxB and that it located primarily to the perinuclear region of the cytoplasm (Fig. 4B), again emphasizing the importance of the subcellular localization of MxB for its antiviral specificity.

Previous studies by Goujon and colleagues assessing the anti-HIV-1 activity of MxA-MxB chimeras also observed a strong correlation between subcellular localization of MxB and anti-HIV-1 function, supporting the evidence for a gatekeeper function of MxB at nucleopores (22). However, our surprising finding that nuclear TMxB blocks HIV-1 does not fit with the proposed gatekeeper function (Fig. 4D). This finding implies that MxB also has the capacity to inhibit HIV-1 replication in the nucleus. To that end, Busnadiego and colleagues previously suggested that MxB has the capacity to inhibit HIV-1 at additional steps of its life cycle (40, 41).

Since the antiviral activities of Mx1 and MxA are associated with a transient interaction with their viral target NP (28, 42–44), it came as no surprise that MxB has the capacity to bind NP (Fig. 5). The fact that MxB not only formed a complex with NP in IAV-infected cells but also in cells ectopically expressing NP indicates that MxB interacts directly with NP, hence not requiring the presence of other viral factors. (Fig. 5A and B). Intriguingly, we observed that wild-type MxB was able to form a stable complex with NP, unlike MxA, which can only stably interact with NP in the dimeric state [variants MxA(R640A) or MxA(L617D)] but not in its wild-type form that exists as a tetramer. The fact that MxB appears to predominantly form dimers (17) most likely explains this apparent difference. The observation that TMxB and NP colocalized throughout the nucleus, with the exception of the TMxB aggregates, suggests that the TMxB-NP interaction does not occur at the large MxB assembly sites. This would fit with the hypothesis that Mx assemblies may represent a nonactive storage form (46).

The capacity of MxB to interact with NP irrespective of its subcellular localization suggests that binding of MxB to NP is not sufficient for blocking IAV replication. It is conceivable that Mx proteins recruit auxiliary factors such as the nuclear export protein UAP56 at distinct subcellular localizations to exert their full antiviral activity and specificity (47–49).

For MxA, the disordered loop L4 (amino acids 533 to 572) plays a decisive role for the recognition of the NP proteins of IAV and Thogoto virus (THOV) as targets (39, 50, 51). In particular, position F561 plays a crucial role for the antiviral activity against IAV and THOV (39, 51). The L4 C-terminal part of MxB shows only limited sequence similarity to the one of MxA (22) and has a 2-amino-acid extension. Nevertheless, it is conceivable that MxB targets the viral NP protein via loop L4, but this remains to be demonstrated.

As expected, expression of TMxB reduced the synthesis of primary viral PB2 mRNA to a similar extent as that of murine Mx1, strongly suggesting that TMxB, like Mx1, interfered directly with viral mRNA transcription by blocking the activity of the viral polymerase complex in the nucleus (Fig. 6) (35, 52). Surprisingly, cells expressing wild-type MxA in the cytoplasm also showed reduced levels of viral primary transcripts. It was previously shown that MxA can inhibit IAV replication at two different steps, namely, (i) during cell entry by blocking incoming vRNPs in the cytoplasm preventing nuclear import of vRNPs (53, 54) or (ii) by interfering with the integrity or transport of newly synthesized NP, a step following primary viral mRNA synthesis (32, 44). It is therefore conceivable that in this experimental setting, overexpression of MxA led to a pronounced block of vRNP import into the nuclei of infected cells, preventing initial viral mRNA synthesis.

Our findings revealed that MxB acquired potent inhibitory activity against IAV when redirected to subcellular compartments where murine Mx1 or human MxA have been shown to block IAV replication. The notion that interaction of MxB with the viral target protein NP is not sufficient for restricting IAV suggests the involvement of auxiliary factors residing at distinct subcellular compartments. It will be interesting to elucidate the contribution of these proteins to the antiviral activities and viral specificities of Mx proteins.

## MATERIALS AND METHODS

### Cell lines.

All cell lines were cultured in Dulbecco’s modified Eagle medium (DMEM) supplemented with 10% fetal calf serum (FCS), 1 mg/ml penicillin/streptomycin and 2 mM GlutaMax (complete DMEM; Thermo Fisher Scientific) at 37°C and 5% CO_2_. HEp-2 (HeLa-derived human epithelial cells), HeLa, and HEK-293T (human embryonic epithelial kidney cells) were purchased from ATCC.

### Plasmids.

As previously described, MxA wild-type cDNA was cloned into pcDNA3.1(+)neo (Invitrogen) (55). MxA(R640A) cDNA in the pcDNA3.1(+)neo plasmid was kindly provided by Georg Kochs (Freiburg, Germany). MxA mutant D250N was generated using the QuikChange II site-directed mutagenesis kit (Agilent). Primers were designed using PrimerX (http://www.bioinformatics.org/primerx/) with the QuikChange protocol.

MxB cDNA with or without an N-terminal FLAG peptide sequence or MxB cDNA with an N-terminal large T-antigen NLS peptide with or without a FLAG peptide were cloned into pcDNA3.1(+)neo (Invitrogen) using the NotI and XbaI restriction sites. Plasmids encoding FLAG-Mx1 or FLAG-Mx1(K49A) were previously described (37). The TMx1-encoding plasmid was described previously (4). Primers were designed using PrimerX with the QuikChange protocol.

gBlocks for untagged MxA-MxB chimera were synthesized by Integrated DNA Technologies (IDT) and cloned into the multiple-cloning site of pcDNA3.1(+)neo. T103A and T151A mutants were generated using the QuikChange II site-directed mutagenesis kit (Agilent). Primers were designed using PrimerX with the QuikChange protocol.

### Viruses.

Infection experiments were carried out using influenza A/Seal/Massachusetts/1/1980 H7N7 (=rSC35M) or A/WSN/1933(H1N1) (=WSN) strains. Infections were performed in phosphate-buffered saline (PBS) supplemented with 0.02 mM Mg^2+^, 0.01 mM Ca^2+^, 0.3% bovine serum albumin (BSA), and 1% penicillin/streptomycin (PBSi) for 1 h at 37°C. Inoculum was removed, cells were washed with PBS, and DMEM supplemented with 20 mM HEPES, 0.2% BSA, 1 mg/ml penicillin/streptomycin and 2 mM GlutaMax (Thermo Fisher Scientific) (piDMEM) was added.

### Immunofluorescence assay.

HEp-2 cells were seeded in 24-well format on glass cover slides. After transfecting with jetPrime (Polyplus-transfection), cells were fixed with 3% paraformaldehyde (PFA) in PBS for 10 min at room temperature and permeabilized with 0.1% or 0.5% Triton X-100 in PBS for 10 min at room temperature. Staining was performed in PBS with 5% goat serum for 1 h at RT with mouse anti-MxA (ɑ-MxA) (hybridoma ab143, 1:5, in house), mouse α-MxB (sc-271527, 1:50; Santa Cruz Biotechnology), mouse α-FLAG M2 (F1804, 1:1,000; Sigma), rabbit α-FLAG (F7425, 1:1,000; Sigma), or mouse α-NP (hybridoma HB65, 1:5, ATCC).

### Minimal replicon reconstitution assay.

The minireplicon assay has been described before (31). Basically, pcDNA3.1(+)neo vectors harboring cDNA sequences of the viral polymerase subunits PB1 and PB2 and PA and viral NP derived from the A/Thailand/1(KAN-1)/2004 (H5N1) strain were transfected into 293T cells. Additionally, the firefly luciferase (FFLuc) reporter plasmid pPOLI-Luc-RT (42) and the constitutively active Renilla reniformis luciferase (RRLuc) plasmid pRL-SV40-*Rluc* (Promega) as a readout and for transfection efficiency were cotransfected using jetPrime (Polyplus-transfection) according to the manufacturer’s instructions. PB1, PB2, and PA (10 ng) and NP, FFLuc, and RRLuc (50 ng) plasmids plus various amounts of plasmids coding for Mx proteins were transfected. For the readout, cells were lysed 24 h after transfection in 60 μl of 1× passive lysis buffer and incubated at room temperature for 15 min on a shaker. A dual-luciferase readout was performed using the Dual-Luciferase reporter assay system (Promega). Lysate (15 μl) was mixed with 45 μl luciferase assay reagent II (LARII) and immediately read on a Perkin Elmer Envision 2104 plate reader for the Firefly luciferase signal; an additional 45 μl of Stop & Glo was added for the *Renilla* luciferase readout.

Western blot staining was performed in 2.5% milk in PBS-Tween for 1 h at RT using rabbit α-FLAG (1:1,000, F7425; Sigma), rabbit α-NP (1:10,000; serum in house), mouse α-actin (1:1,000, sc-47778; Santa Cruz Biotechnology), and mouse α-Mx (1:20, in house, ab143).

### Plaque assay.

293T cells were transfected with plasmids coding for MxA and MxB variants or GST or mCherry as controls. At 24 h postinfection, the cells were infected with rSC35M or WSN at a multiplicity of infection (MOI) of 0.01 for 24 h. Supernatant was harvested, and MDCK cells were subsequently inoculated with diluted supernatant (from 10^−1^ to 10^−6^ in 1:10 dilution steps) for 1 h at RT. Inocula were aspirated, and a 1% Avicel in H_2_O overlay was applied. At 28 h postinfection, the Avicel was aspirated and the cells were fixed for 10 min with 3% PFA and stained for 15 min with 0.1% crystal violet in 10% methanol.

### CellTiter-Glo assay.

293T cells were transfected and at 48 h posttransfection, half the volume of the medium was aspirated. An equal volume (as aspired medium) of Cell-Titer-Glo (Promega) reagent was added to the wells, incubated for 2 min at room temperature on a shaker and for an additional 10 min without shaking, and luminescence was measured on a Perkin Elmer Envision 2104 plate reader.

### Coimmunoprecipitation.

HEp-2 cells were transfected with ViaFect (Promega) according to the manufacturer’s instructions. At 24 h posttransfection, cells were lysed in 300 μl of lysis buffer (20 mM Tris-HCl [pH 7.5], 150 mM NaCl, 5 mM MgCl_2_, 50 mM NaF, 1 mM Na_3_VO_4_, 1% NP-40, 50 mM β-glycerophosphate, 100 nM iodoacetamide, and 1× Roche cOmplete protease inhibitor cocktail) in the dark on ice for 30 min. Lysates were homogenized using QiaShredder columns (Qiagen).

In the case of a coimmunoprecipitation (co-IP) experiment from infected cells, HeLa cells were infected at 24 h after transfection (see above) with WSN at an MOI of 5 for 6 h.

Aliquots (100 μl) of each lysate were incubated with the antibody α-NP (in-house, 50 μl, HB65, hybridoma) at 4°C overnight on a rotating wheel. In the infection IP, lysates were incubated 5 h with antibodies (either α-NP [see above] or 1 μg mouse α-FLAG [F1804; Sigma]). Dynabeads protein G magnetic beads (Thermo Fisher Scientific) (20 μl per sample) were preadsorbed overnight at 4°C on a rotating wheel using untransfected HEp-2 cell lysate. Preadsorbed beads were washed once with lysis buffer and incubated with the lysates for 1 h at 4°C on a rotating wheel. Beads were washed five times with 0.5 ml lysis buffer. Beads were briefly vortexed between the washing steps. Proteins were eluted from the beads with 20 μl of 1× Laemmli buffer at 95°C for 10 min. Lysates were analyzed by Western blotting using rabbit α-NP (1:10,000; in-house), mouse α-FLAG (F-3165, 1:1,000; Sigma), rabbit α-FLAG (F7425, 1:1,000; Sigma), rabbit α-actin (A2103, 1:1,000; Sigma), mouse α-MxB (271527, 1:500; Santa Cruz Biotechnology), or rabbit α-MxA (H00004599_D01P, 1:1,000; Abnova).

### Luciferase assay with VSV-G-pseudotyped HIV-1 NL-Luc reporter virus.

HeLa cells were transfected with 75 ng of expression plasmid and 425 ng of puromycin resistance plasmid. At 24 h posttransfection, cells were infected with the NL-Luc virus stock (1:27 dilution) in Opti-MEM reduced serum medium (Life Technologies) plus 8 μg/ml Polybrene (Sigma-Aldrich) and 1 μg/ml puromycin (InvivoGen) for 90 min at 37°C and 5% CO_2_. The inoculum was removed, and the cells were washed with PBS and incubated at 37°C and 5% CO_2_ for 48 h in growth medium (complete DMEM) with 20% FCS plus 1 μg/ml puromycin to ensure survival and expression of reporters in transfected cells only. Luciferase readout was performed with the BrightGlo luciferase assay system (Promega) on an Envision 2104 plate reader (Perkin Elmer) according to the manufacturer’s instructions.

### qRT-PCR.

293T cells were transfected using jetPrime (Polyplus-transfection) according to the manufacturer’s instructions. At 24 h posttransfection, cells were pretreated for 1 h with 100 μg/ml cycloheximide (CHX) or dimethyl sulfoxide (DMSO). Cells were inoculated with virus in PBSi for 1 h at 37°C with rSC35M or SFV V42 at an MOI of 5. The inoculum was aspirated and piDMEM was added. At 5 h postinfection, the medium was removed and cells were lysed with 200 μl TRIzol reagent (Thermo Fisher Scientific) per well of a 24-well plate. After the addition of 40 μl of chloroform, the samples were mixed thoroughly by inverting the tubes several times and then centrifuged at 13,000 × *g* at 4°C for 15 min. The resulting upper aqueous phase (∼120 μl) was transferred to a new tube, and RNA extraction was subsequently performed using the RNeasy minikit (Qiagen) starting with the addition of 1 volume of 70% ethanol (EtOH). cDNA synthesis was performed with 1 μg RNA using SuperScript III reverse transcriptase (Thermo Fisher Scientific) and random primers. The quantitative PCR (qPCR) was performed with 2.5 μl of the 1:10 diluted cDNA and EvaGreen fluorescent nucleic acid dye in a total volume of 20 μl. The cycling conditions were as follows: initial denaturation, 95°C for 5 min; denaturation, 95°C for 15 s; annealing and extension, 60°C for 60 s. A dissociation step starting at 55°C was added in the end to confirm the specificity of the primers. The primer sequences are as follows: rSC35M PB2 forward primer, 5′-GCAATGGGCTTGAGGATT; rSC35M PB2 reverse primer, 5′-CAATCTCCTGGTTGCCTT; GAPDH forward primer, 5-CTGGCGTCTTCACCACCATGG; and GAPDH reverse primer, 5-CATCACGCCACAGTTTCCCGG. The fold change of PB2 mRNA normalized to GAPDH mRNA was calculated according to Pfaffl (56). In short, first, the threshold cycle (Δ*C_T_*) of a given sample was calculated [*C_T_*(CHX) − *C_T_*(DMSO)], then ΔΔ*C_T_* was assessed [Δ*C_T_*(Mx) − Δ*C_T_*(mCherry)], and the final values for plotting were shown as 2^−ΔΔCT^ (in percentages).
